# Poster Session II - A292 ASSESSING ULCERATIVE COLITIS DISEASE ACTIVITY DURING PREGNANCY: A RETROSPECTIVE COHORT STUDY OF FECAL CALPROTECTIN, C-REACTIVE PROTEIN, AND THE PARTIAL MAYO SCORE.

**DOI:** 10.1093/jcag/gwaf042.292

**Published:** 2026-02-13

**Authors:** K Kecskemeti, H Nabavian, S Eisen, V Srikanth, K O’ Connor, V W Huang

**Affiliations:** Faculty of Medicine, University of Toronto, Toronto, ON, Canada; Faculty of Medicine, University of Toronto, Toronto, ON, Canada; Faculty of Medicine, University of Toronto, Toronto, ON, Canada; Faculty of Medicine, University of Toronto, Toronto, ON, Canada; Faculty of Medicine, University of Toronto, Toronto, ON, Canada; University of Toronto, Toronto, ON, Canada

## Abstract

**Background:**

Ulcerative Colitis (UC) often manifests during patient’s early adulthood which coincides with reproductive years for women. Monitoring disease activity during pregnancy is an essential yet understudied area of clinical research. Pregnancy is known to alter UC symptoms and disease activity through hormonal and fetal development changes.

**Aims:**

This study aims to assess the concordance between biochemical markers fecal calprotectin (FCP) and C-reactive protein (CRP) and the partial Mayo (pMayo) clinical score. We aim to investigate pregnancy-specific FCP thresholds for defining clinically active disease in pregnant IBD patient at each trimester.

**Methods:**

We identified all pregnant patients with UC who were seen at the Mount Sinai Hospital Pregnancy IBD clinic from January 2017 to December 2022. We analyzed demographic characteristics including age and parity. Biochemical markers FCP and CRP were recorded at each trimester. We collected clinical pMayo scores which were recorded during clinic visits at each trimester and gathered from the outpatient EMR. We defined active clinical disease if a patient had an pMayo ≥ 2, or a clinician-documented impression of active disease. Spearman’s rho tests were performed to analyze the correlation between the pMayo scores, FCP and CRP for each trimester. We completed a bivariate assessment with Chi-Squared tests to determine FCP cut-offs for patients with active and inactive clinical disease activity during pregnancy.

**Results:**

A total of 119 pregnant patients with UC were included in the study. The mean maternal age at conception was 32.9 ± 3.7 years. First-time pregnancies accounted for 69 cases (57%). Table 1 documents the Spearman’s correlation coefficients between pMayo scores and FCP values. Trimesters 2 and 3 had a significantly positive correlation coefficient. There was no linear correlation between CRP levels and pMayo scores at any trimester (Table 2). To evaluate FCP thresholds for clinical activity during pregnancy, various values were investigated. A threshold of 150 µg/g was significantly for Trimesters 2 and 3, however not for Trimester 1.

**Conclusions:**

For pregnant patients with Ulcerative Colitis the partial Mayo score is concordant and positively correlated with FCP levels later in pregnancy (trimester 2 and 3). This relationship was not observed with CRP levels. A threshold FCP 150 µg/g was statistically significant, however not in trimester 1. Future research should be pursued to determine the impact of these thresholds on maternal and fetal pregnancy outcomes in patients with IBD.

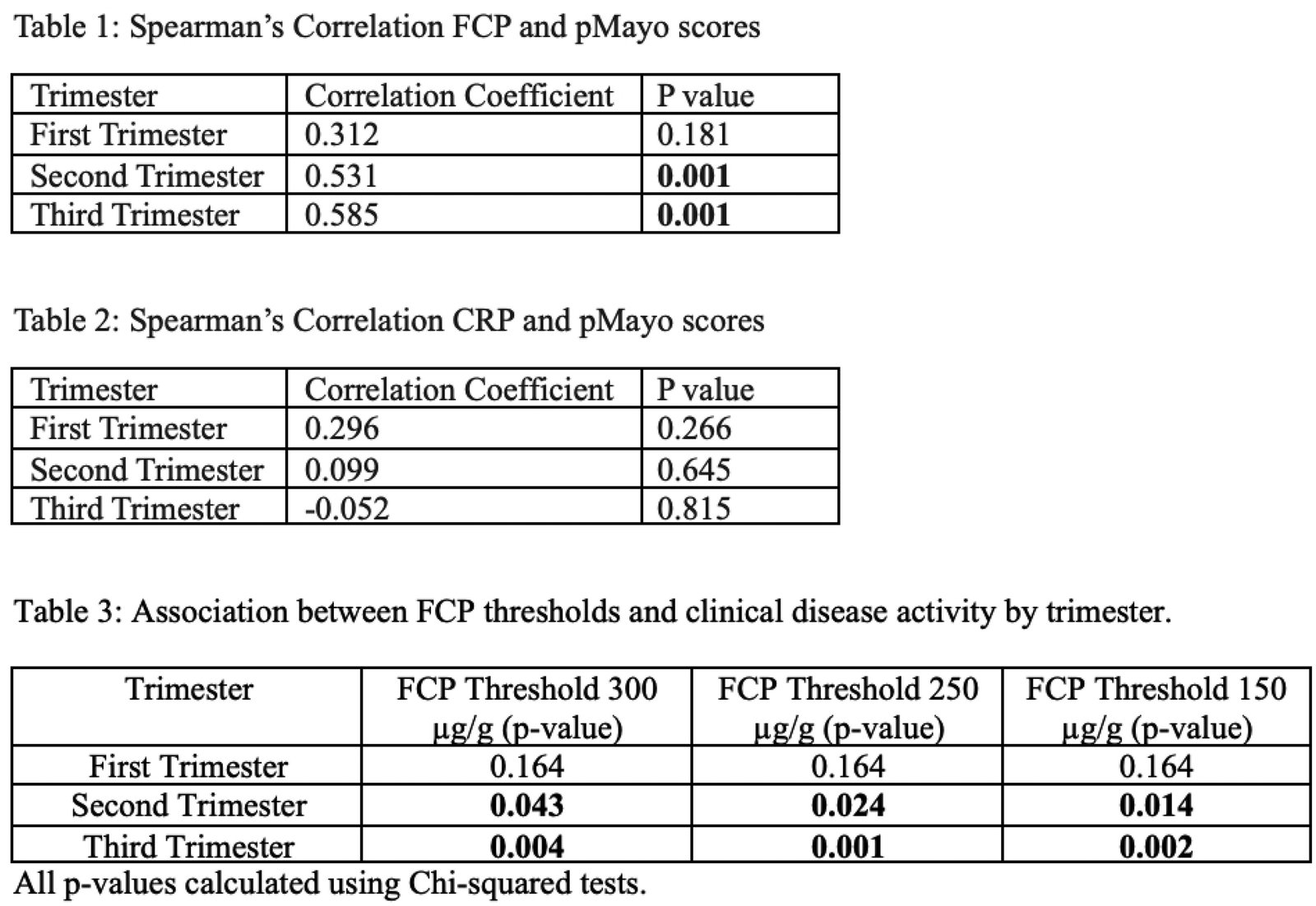

**Funding Agencies:**

NoneMount Sinai Hospital Resident Research Scholarship

